# Deciphering animal development through proteomics: requirements and prospects

**DOI:** 10.1186/1477-5956-6-21

**Published:** 2008-07-24

**Authors:** Wolfgang E Reintsch, Craig A Mandato

**Affiliations:** 1 Department of Anatomy and Cell Biology, McGill University, 3640 University Street, Montreal, Quebec, H3A 2B2, Canada

## Abstract

In recent years proteomic techniques have started to become very useful tools in a variety of model systems of developmental biology. Applications cover many different aspects of development, including the characterization of changes in the proteome during early embryonic stages. During early animal development the embryo becomes patterned through the temporally and spatially controlled activation of distinct sets of genes. Patterning information is then translated, from gastrulation onwards, into regional specific morphogenetic cell and tissue movements that give the embryo its characteristic shape. On the molecular level, patterning is the outcome of intercellular communication via signaling molecules and the local activation or repression of transcription factors. Genetic approaches have been used very successfully to elucidate the processes behind these events. Morphogenetic movements, on the other hand, have to be orchestrated through regional changes in the mechanical properties of cells. The molecular mechanisms that govern these changes have remained much more elusive, at least in part due to the fact that they are more under translational/posttranslational control than patterning events. However, recent studies indicate that proteomic approaches can provide the means to finally unravel the mechanisms that link patterning to the generation of embryonic form. To intensify research in this direction will require close collaboration between proteome scientists and developmental researchers. It is with this aim in mind that we first give an outline of the classical questions of patterning and morphogenesis. We then summarize the proteomic approaches that have been applied in developmental model systems and describe the pioneering studies that have been done to study morphogenesis. Finally we discuss current and future strategies that will allow characterizing the changes in the embryonic proteome and ultimately lead to a deeper understanding of the cellular mechanisms that govern the generation of embryonic form.

## Introduction

Embryogenesis, the formation of a complete organism from an undifferentiated egg, has fascinated observers since Aristotelian times and experimental approaches to unravel the mechanisms behind embryogenesis date as far back as the late 19^th ^century [1]. Early embryologists used ablation and transplantation techniques to manipulate embryos and it was soon realized that developing embryos have a high capacity to self-regulate. This was first demonstrated by Hans Driesch, who, by separating the two first blastomeres of see urchin embryos, demonstrated that each blastomere has the capacity to form a complete, half-sized organism [2]. The work of Driesch and other embryologists found its culmination in the definition of the "organizer" by Spemann and Mangold. They showed that the transplantation of a small region from the dorsal side of an early gastrula embryo into the ventral side of a host embryo results in the formation of a Siamese twin with a complete secondary axis [3]. Both donor and host cells contributed to this secondary axis indicating that the transplant induced surrounding cells to change from ventral to more dorsal fate and to participate in the necessary movements to form an elongated axial structure. For the prove of embryonic induction Hans Spemann received the Nobel Prize of Medicine in 1935. The question of what constitutes such inducing signals in the embryo (patterning) and how they control cell behavior to form the shape of the embryo (morphogenesis) has remained at the core of developmental biology since these seminal experiments.

### The molecular basis of patterning

During the early period of experimental embryology, fundamental concepts have been formulated despite the lack of any knowledge about the molecular basis of development. One of the most influential theories was certainly the proposal of morphogens as inducers of patterning [4,5]. Morphogens are defined as factors that, diffusing from a local source, generate a gradient that determines the cell fate of surrounding tissues in a concentration-dependent manner. The search for the molecular basis of patterning received an enormous boost with the execution of the first large scale mutational screen for defects in early Drosophila development. This led for example to the molecular description of the first morphogen, Bicoid, a transcription factor that forms a concentration gradient in the anterior half of the syncytial fly embryo and defines different anterior structures [6,7]. Many other model systems, like e.g. frog, nematode, zebrafish and mouse, have been explored since then and have contributed to our current understanding of patterning processes (e.g. [8-10]). Today we know that patterning and the fascinating ability of embryos to self-regulate and regenerate is based on a complex interplay between signaling pathways that relies mainly on secreted signaling molecules, their antagonists and the local activity of transcription factors [11]. The exponential increase in knowledge about the molecular basis of patterning is intimately linked to the rise of molecular biology. The development of efficient sequencing, in situ hybridization, RNAse protection and RT-PCR has allowed determining the spatial and temporal distribution of mRNA encoding relevant factors in the embryo and in isolated tissues. Molecular cloning has allowed for the generation of deletion constructs to define functionally relevant domains in proteins, for the design of dominant negative and constitutively active constructs and for the use of reporter constructs to study the activity of promoter regions. In addition, the discovery of RNAi and the development of new, modified antisense constructs have added new techniques to the molecular "tool box" that have become invaluable to study gene function [12,13].

It appears now that in several species, including the "classic" model systems Drosophila melanogaster and Xenopus laevis, a reasonably "complete" list of genes that are involved in early pattern formation has emerged. This undertaking has become even more feasible in recent years, since the genomes of several model animals have been completely sequenced, including fruit fly, nematode and sea urchin, and other genomes are close to completion and/or large cDNA and EST databases have been built [14-19]. Therefore in several species the resources and the tools are available to characterize the spatial and temporal expression of every gene. The systematic characterization of expression patterns and regulatory interactions between patterning genes is well underway [15,20-22], shifting the focus of this research now to the analysis of the regulatory networks that are formed by these genes [23,24] and the mathematical modeling of patterning events in early embryos [25]. Taken together, during the last thirty years embryology has made huge strides to elucidate the molecular basis of pattern formation and thereby to answer questions of embryology that have been raised a century ago. However, it has also become evident that the systematic approaches (e.g. mutagenesis, overexpression and knock down screens) used so successfully to study pattern formation are not sufficient to resolve questions of morphogenesis in a similar manner.

### The molecular basis of morphogenetic movements

To study morphogenesis essentially means to ask how cells and tissues translate the positional information they have received into regional specific cell behaviors to give the embryo its defined shape. In animals, the first global cell rearrangements occur as gastrulation is initiated. Gastrulation is defined as the internalization of the prospective endoderm and mesoderm into the embryo and, while different species use different means and mechanisms to achieve internalization, the result is always the same: the endoderm forms the inner layer, the ectoderm remains on the outside and the mesoderm is located in between. After gastrulation, the three germ layers are in close apposition to each other; but they are clearly separated and mixing between the different layers rarely occurs, indicating that these cells can distinguish "similar" cells from "different" cells. This ability was demonstrated in dissociation and reaggregation experiments. Cells from early amphibian embryos can easily be separated by removal of calcium from the culture medium. Mixing of cells from the different germ layers and restoring of the calcium levels will lead first to a ball of mixed cells, but then cells will not only reaggregate according to their germ layer affiliation, but also assume the same position as in the embryo: ectoderm on the outside, mesoderm next and endoderm on the inside. These classical experiments led to the proposal of differential affinity between germ layer cells and later to the concept of differential adhesion to explain the ability of cells of different tissues to remain separate from each other [26-29]. One very important family of cell-cell adhesion molecules is constituted by transmembrane molecules of the cadherin family [30]. However, how their activity is differentially regulated between germ layers and later between developing tissues is to a large extent unclear. For example, C-cadherin is absolutely required for cell-cell adhesion in the early Xenopus embryo [31,32] and its adhesive strength and dynamics are tightly regulated during gastrulation to facilitate cell movements and the separation of the germ layers. While some of the signals that change C-cadherin activity have been characterized in this context, it is still unknown how these changes are mediated on the mechanical level [33-36].

Likewise, our understanding of the molecular mechanisms that drive the cellular movements and cell shape changes during embryogenesis is still very fragmented. In several species gastrulation movements have been characterized in great detail and employed to develop general models of coordinated cell movements and shape generation [37-41]. These movements can often be correlated with the expression of certain patterning genes, but the downstream signals that regulate cell behavior and the means by which they modify mechanical attributes of cells are mostly unknown. The actin cytoskeleton for example is certainly involved in cell movements that require protrusive and contractile activity and the enormous progress that cell biologists have made in identifying the protein components that provide the basis of cytoarchitecture has sparked new interest in characterizing the role of these proteins in the embryo [42-45]. However, in general changes in the architecture, dynamics and contractility of the cytoskeleton appear to rely to a large extend on signaling, posttranslational modifications and differential use of a set of accessory proteins and much less on changes in gene expression [46-49]. Many cytoskeletal proteins have also essential functions in other processes like e.g. cell division and endo-exocytosis [50,51], which might render it often very difficult, if not impossible, to interpret the effects of a broad interference with their activity. In addition, the control of translation and posttranslational modifications of proteins provide additional layers of regulation that might influence the abundance, splicing variants, subcellular localization and activity of a given protein in a cell. Nevertheless, progress has been made for example to bridge the gap between patterning signals and cell shape changes [52-54] or to elucidate the role of actin regulators [44,55,56] in specific morphogenetic movements. However, it appears that the molecular basis of cell behavior is difficult to decipher systematically by the means of molecular and genomic biology (e.g. mutational screens and gene expression analysis). Proteomic techniques provide in principle the right tools to directly characterize the changes in concentration, posttranslational modification and complex composition of sets of proteins during development and systematic proteomic approaches will hopefully allow to gain further insight into the molecular mechanisms that drive morphogenesis.

### Analyzing developmental processes with proteomic tools

Proteomic approaches have already strongly contributed to many research areas in the medical sciences and in biology. Such areas include for example the study of cellular organelles [57-60] and protein interaction networks [61-63], certain aspects in cancer research [64,65] and the study of synapses in neurobiology [66-68]. In recent years proteomic techniques have also become employed in several model systems of developmental biology, in a diverse array of contexts. A selection of reports on "developmental proteomics" is summarized in Table 1. Embryogenesis -in molecular terms- is reflected in the temporal and spatial changes of protein presence and activity. Several studies have been performed to compare levels and/or isoform shifts of proteins between different stages of development in zebrafish, Drosophila, mouse and chick [69-74] or to characterize protein differences between different embryonic tissues at the same point of development [75-77]. In almost all of these studies the proteome has been characterized using 2D-gel based protein separation and subsequent mass spectroscopy. This approach is relatively straight forward and allows detecting changes in protein quantities as well as shifts in isoform distribution directly on the gel. Apart from general characterizations of embryonic proteomes, 2D-gel approaches have also been used to detect protein differences after specific signaling events. One example is the comparison of egg proteins from sea urchin before and after fertilization. In this case the combination of protein and phospho-specific stains has allowed to identify several proteins that change in abundance and phosphorylation state within minutes after fertilization [78]. Another example is the characterization of protein differences after Thyroid hormone treatment in Rana tadpoles to study metamorphosis, which has, among other things, led to the identification of a novel larval keratin as a target of Thyroid-mediated signaling [79]. However, a 2D-gel approach has certain limitations: low abundance proteins might not be detected since the amount of sample that can be loaded on a 2D-gel is limited and the cellular concentration of proteins varies widely [80,81]. Highly abundant proteins will also mask close by or overlapping weaker protein spots. In addition, certain types of proteins have properties (e.g. high hydrophobicity) that make them difficult or even impossible to be resolved in a 2D-gel approach. This can for example lead to an underrepresentation of integral membrane proteins [81,82]. An alternative method is provided by "peptide-centric" proteomics, were the proteins in a given sample are digested first, yielding in general more soluble fragments, and the resulting peptide-mix is fractionated and analyzed by mass spectroscopy [83]. In a recent study in zebrafish embryos, both methods were employed in parallel. Interestingly, only about 30% of the characterized proteins appeared in both approaches, indicating that the two methods might rather complement each other [70]. In general, each approach to analyze an embryonic (or other organismal) proteome appears to yield only a subset of proteins at the present time. More complete inventories of an embryonic proteome at a given stage of development can certainly be obtained through a combination of different approaches, including initial subfractionation/enrichment steps to reduce the complexity of a sample before protein identification [84]. On the peptide side the complexity can be reduced by selection of a limited number of representative peptides to be analyzed per protein [85,86]. On the protein side the sample can for example be fractionated according to protein size, by one-dimensional gel electrophoresis, before digestion [57]. Other fractionation techniques allow to separate proteins according to their spatial distribution in a cell (e.g. organelle fractions) or according to their posttranslational modifications (e.g. phosphorylation) [59,87,88]. Such approaches have been used already to isolate specific subpopulations of proteins from embryos. Several studies have been performed to biochemically isolate and then characterize phosphorylated proteins from Drosophila, mouse and zebrafish embryos [89-92]. The large phosphopeptide databases generated from such studies provide new insights into signaling pathways that are active during development. Other studies include for example the isolation of matrix vesicles during chick embryo bone formation [93]. These organelles are important mediators of bone mineralization. 126 organelle proteins were identified, several of which were previously unknown. In the nematode C.elegans biochemical purification of chondroitin proteoglycans and subsequent mass spectroscopy led to the characterization of 9 new proteoglycan core proteins with no apparent sequence homology to chondroitin proteoglycans in other species [94]. Simultaneous RNAi-based knock down of two of these proteins results in multinucleated single-cell embryos, indicating an essential role for chondroitin proteoglycans during cytokinesis. Results like these demonstrate how the combination of biochemistry to isolate a cellular organelle or protein subpopulations and mass spectroscopy can provide powerful tools to dissect developmental processes that would have been much more difficult if not impossible to analyse otherwise.

**Table 1:** Use of proteomics in developmental studies.

**Model**	**Embryos, Stage(s)**	**Characterization**	**Results**	**Source**
Zebrafish	72 (hatching) and 120 hpf (larvae)	2D LC ESI-MS/MS Temporal proteome	1112 pr. (72 hpf) 867 pr. (120 hpf)	[70]
Zebrafish	72 (hatching) and 120 hpf (larvae)	2D PAGE MALDI TOF/TOF Temporal proteome	348 pr. (72 hpf) 317 pr. (120 hpf)	[70]
Zebrafish	24 hpf	SCX-TiO2-LC MS/MS Phosphoproteome	604 phoshorylated pr.	[90]
Zebrafish	24 hpf wild-type and Fyn/Yes knock down embryos	SCX-TiO_2_-LC MS/MS Phosphoproteome	141 pr., differentially phosphorylated	[91]
Zebrafish	7 hpf (early gastrula)	DIGE LC MS/MS Tissue proteome	35 differentially expressed pr. between mesoderm and ectoderm	[75]
Zebrafish	6h-1 week pf (pregastrula-larvae stage)	2D PAGE MALDI TOF/TOF Temporal proteome	55 differentially expressed pr.	[69]
Zebrafish	2dpf, cloche mutant and wild-type	2D PAGE MS/MS (-)	γ-cristallin downregulated in cloche mutants	[122]
Drosophila	0 h–24 h (combined)	SCX-IMAC-LC MS/MS Phosphoproteome	2702 phosphorylated pr.	[89]
Drosophila	Embryos (0–22 h), adult heads	SCX LC MS/MS Temporal proteome	660 respectively 780 pr., 307 pr. in both stages	[74]
Drosophila	gastrula-stages	DIGE MALDI TOF/TOF Tissue proteome	37 differentially expressed pr. between lateral and ventral tissue	[76]
C.elegans	Mixed stages	SCX LC-MS/MS Purified proteoglycans	9 novel chondroitin proteoglycans	[94]
C.elegans	embryo, L1-L4 larvae, adult	DIGE MALDI TOF Temporal proteome	165 pr. expression profile	[123]
chick	Matrix vesicles	SDS-PAGE LC MS/MS Organelle proteome	126 pr.	[93]
chick	ED 7 and 11 retina tissue	2D PAGE MALDI TOF Temporal proteome	13 pr., differentially expressed	[72]
chick	Stage 29 (6 days)	2D PAGE MALDI TOF Tissue proteome	105 pr.	[124]
Rat	ED 11.5 embryo vs. Yolk sac membrane	2D PAGE MALDI MS/MS Tissue proteome	430 tissue specific pr. spots	[77]
mouse	Brain tissue ED 9.5, 11.5, 13.5	DIGE ESI MS/MS Temporal proteome	195 pr. differentially regulated	[125]
mouse	Brain tissue ED 16 and postnatal	2D PAGE MS Tissue proteome	~10 pr.	[71]
mouse	Brain tissue ED 16,5	SCX LC MS/MS Phosphoproteome	546 phosphorylation sites	[92]
mouse	ED 8.5, 9.5, 10.5 neural tube closure	2D PAGE ESI MS/MS Temporal proteome	14 pr. upregulated at ED 10.5	[73]
Sea urchin	Egg, pre-post-fertilized	2D PAGE MS/MS Phosphoproteome	94 pr. show changes after fertilization	[78]
Artemia	Diapause	2D PAGE LS MS/MS Temporal proteome	33 pr.	[126]
Artemia	Postdiapaused cysts (0–20 h)	2D PAGE MALDI TOF/TOF Temporal proteome	75 pr. differentially expressed	[127]
Rana catesbeiana	Tadpole (tailfin)	2D-PAGE or iTRAQ-MS/MS Temporal proteome	17 pr. differentially expressed after Thyroid treatment	[79]

A summary of published results on the use of proteomics in different model systems of developmental biology (pf = postfertilization, pr = proteins)

### Proteomic approaches to study morphogenesis

During embryogenesis, discrete regions in the embryo display specific morphogenetic activities. The sum of these activities produces the final shape of the embryo. Regionalization of morphogenetic behavior poses an important challenge for the application of proteomic tools, mainly because sufficient amounts of a given tissue -that undergoes a specific movement- have to be obtained for subsequent analysis. Tissue formation and thereby the related cell behavior is under the control of upstream patterning signals and, to obtain large amounts of starting material, the manipulation of such signals has been employed to obtain mutant embryos that are either deficient or enriched in a certain tissue.

In Drosophila gastrulation begins with the internalization of mesodermal precursor cells on the ventral side of the embryo. This movement is initiated by shape changes of ventral cells that lead to the formation of a furrow. To study changes in the protein composition of ventral cells during this period of development the group of Jonathan Minden compared mutant embryos which are strongly ventralized to embryos where the ventral cells have adopted a lateral fate [76]. By difference gel electrophoresis (DIGE), they identified a total of 1315 protein spots, 105 of which showed differences in expression or isoform distribution. They were able to determine the identity of 65 differentially expressed protein spots, originating from 37 unique proteins [76]. The largest groups of isolated proteins were comprised by metabolic enzymes and proteases. However, in addition several cytoskeleton and membrane associated proteins were found to be differentially regulated, providing a starting point for a more detailed analysis.

A second study compared gastrulation stage zebrafish embryos that consisted mainly of cells of ectodermal respectively mesendodermal character [75]. In zebrafish, mesendoderm induction and the subsequent ingression of these cells during gastrulation depend on TGF-β-like Nodal signals. Suppression of this signal, in this study by using one-eyed-pinhead mutant embryos, produces "ectodermal" embryos [95], while overexpression of Nodal results in "mesendodermal" embryos [75,96]. Using DIGE, 36 differentially expressed proteins were identified, including several cytoskeletal proteins. Among these proteins was Ezrin, a member of the ERM-family of proteins that links transmembrane proteins to the actin cytoskeleton [97]. The activity of these proteins is regulated by phosphorylation and closer analysis of Ezrin in the gastrulating zebrafish showed that it becomes preferentially phosphorylated in the mesendoderm. In addition, antisense-mediated knock down of Ezrin resulted in reduced cell migration of mesendodermal precursors during gastrulation, indicating that proper expression of Ezrin is important for these movements.

Another possibility is to interfere specifically with signals that have been found to be involved in the control of tissue movements and to identify thereby downstream signaling events. This approach has been used to study Fyn/Yes dependent signal transduction during zebrafish axis formation [91]. During vertebrate gastrulation, cells converge towards the dorsal midline, which leads to an elongation of the anterior-posterior axis and the formation of the characteristic elongated shape of the postgastrula embryo. This behavior has been termed convergence and extension [98]. Convergence and extension movements occur also during other stages of development and organogenesis to form elongated structures [40]. The non-canonical Wnt pathway has been found to be central to the regulation of this movement during gastrulation and several downstream effectors have been characterized, including the small GTPases Rho, Rac and Cdc42 [99-101]. In addition, two members of the Src-family of kinases, Fyn and Yes appear to converge on RhoA and knock down of these two proteins via an antisense approach leads to a phenotype similar to the phenotype after interference with the non-canonical Wnt pathway [102]. To further characterize the effect of the Fyn/Yes knock down on signaling during this essential morphogenetic movement, the group of den Hertog compared the phosphoproteome of Fyn/Yes knock down embryos to the phosphoproteome of wild type embryos [91]. Using an automated titanium dioxide-based LC-MS/MS set-up to enrich for phosphorylated peptides [90], they were able to identify 348 phosphoproteins. 69 of these proteins were found to be downregulated and 72 proteins upregulated in the Fyn/Yes deficient embryos. Several of the differentially regulated proteins found in this screen have already -directly or indirectly- been implicated in the regulation of gastrulation movements and/or in the reorganization of the cytoskeleton. In addition this study provides many new leads to further characterize the signaling network that regulates convergence and extension movements during development.

The last decades have provided a large pool of mutants and antisense tools to manipulate patterning and morphogenetic events in the embryo. Combined with the continuous improvements of proteomic techniques and the increasing availability of proteomic facilities this will certainly lead to an increase in comparative proteome studies of mutant or knock down embryos. In addition, comparative studies on isolated tissues of an embryo might also be possible. Most notably, amphibian embryos like Xenopus laevis are known for the ease with which relatively high amounts of different tissues can be manually isolated. Manual isolation has been employed in many different contexts, e.g. to construct tissue specific libraries, to compare expression of marker genes or for antibody-based comparison of protein levels [103-105] and should also be suitable for proteomic studies. Another technique that allows to collect different tissues from embryos is laser-assisted microdissection (LAM) [106]. This method allows to cut specific regions from tissue sections and can provide sufficient material for proteomic analysis [107,108]. So far LAM has been mainly used in clinical applications. However it has already been applied to isolate tissue fragments from Xenopus laevis embryos for subsequent mRNA isolation, demonstrating its applicability during early stages of development [109]. Another exciting technical advancement for tissue specific protein identification is the development of imaging mass spectrometry (IMS). This technique allows the direct detection of proteins by mass spectroscopy on histological sections [110]. In principle this method already provides the means to compare the distribution of multiple proteins between different tissues without their preceding isolation. While currently still lacking the necessary resolution and sensitivity for most applications in embryology, it has transformed the mass spectrometer into a veritable protein microscope and with frequent technical improvements and increasing availability IMS might become a valuable tool to study protein distribution in the embryo [111].

The examples cited above illustrate how comparative studies can provide novel candidates for proteins that participate in the regulation and execution of morphogenetic movements. 2D-gel-based approaches were used most commonly for good reasons, but it might be beneficial to additionally use peptide-based approaches to detect changes in protein concentration that are underrepresented in 2D-gels [80-82]. This becomes especially useful since it appears that the correlation between mRNA and protein levels is weak [112,113] and therefore peptide based approaches might reveal protein differences that are not detectable through genomic approaches. In this regard it would be interesting to determine the relative importance of transcriptional versus translational/posttranslational regulation on protein concentration and function in the embryo. Selective isolation of phosphoproteins in the context of morphogenetic movements highlights the possibility to characterize specific subsets of proteins and to link thereby signaling to the regulation of downstream effectors [91]. With increasingly detailed annotation of proteins and the continuous development of protein-protein interaction and signaling pathway databases [114,115] it will hopefully soon be possible to reconstruct tissue and/or stage specific signaling networks from such data. Other subsets of relevant proteins that could possibly be isolated through subfractionation approaches are for example membrane-bound proteins [64,116]. Cells are in constant communication with their environment and differential expression or activity of surface proteins is very likely involved in many processes of morphogenesis, including for example cell adhesion, directed cell and tissue movement or tissue separation [117-119]. Isolation of such proteins will provide new candidates to link external signals to internal changes in the mechanical properties of cells. Furthermore, improved affinity purification methods allow isolating protein complexes within signaling networks and, combined with subsequent mass spectroscopy, to identify novel complex associated proteins [120,121]. Potentially, such methods can also be used to monitor changes in complex compositions in comparative studies of different stages/tissues or mutated versus wild type embryos and thereby allow further insight into the regulatory networks that controls cell behavior.

## Conclusion

Within just a few years proteomic techniques have been used in a variety of model organisms of developmental biology and in applications ranging from the development of species specific protein databases down to the isolation and identification of single proteins of interest. These pioneering studies demonstrate the usefulness of proteomic approaches and with increasing availability of proteomic facilities and technical expertise, such approaches offer exciting possibilities in many areas of embryology. These new possibilities -we believe- will strongly influence the study of morphogenesis. So far our understanding of the driving forces behind morphogenetic movements is largely based on detailed descriptions of changes in cell shape and protrusive activity as well as explantation/ablation techniques to elucidate their relative contribution to the forces that form the embryo. However, cell biology has already made incredible progress in the characterization of the protein components that determine the mechanical attributes of a cell, its internal architecture and its external interactions. Drawing from the immense wealth of cell biological studies, morphogenetic studies have also been extended to the investigation of some of the molecular regulators of cell mechanics. Proteomics provide now the means to systematically study the regulatory events that link patterning signals to the structural changes that determine tissue specific cell behavior. In the short term this will provide new candidate regulators of morphogenesis. In the longer term, the continuing improvements in proteomic techniques and data analysis and presentation will provide a more comprehensive picture of time and tissue specific protein composition and posttranslational modifications. This will allow for a systematic analysis of the active signaling networks in a given tissue at a given time point and form the necessary basis for a multidisciplinary approach that includes e.g. biomechanics and *in vivo *imaging, to decipher how these signals are coordinated to produce the forces that shape a complete organism from an undifferentiated ball of cells.

## Competing interests

The authors declare that they have no competing interests.

## Authors' contributions

WER and CAM co-wrote the manuscript and approved the final version.
